# Active Vaccination Strategies for Alzheimer's Disease: An Expanded Review

**DOI:** 10.1002/eji.70250

**Published:** 2026-08-17

**Authors:** Antonella Comitato, Antonino Neri, Andrea Cossarizza

**Affiliations:** ^1^ Scientific Directorate Azienda USL‐IRCCS di Reggio Emilia Reggio Emilia Italy; ^2^ Università di Modena e Reggio Emilia Modena Italy

**Keywords:** β‐amyloid, Aβ, active immunotherapy, Alzheimer's disease, disease‐modifying therapy, MultiTEP platform, SupraAntigen, Tau protein, UBITh, vaccine development

## Abstract

Alzheimer's disease (AD) is pathologically defined by extracellular β‐amyloid (Aβ) aggregates and intracellular hyperphosphorylated Tau, which collectively drive synaptic failure, neuroinflammation and progressive neuronal degeneration. Since current therapies provide predominantly symptomatic relief without halting disease progression, the development of robust disease‐modifying interventions remains a critical unmet medical need. Here, we discuss the most recent advances in active immunisation strategies targeting pathological Aβ and Tau species and their potential to meaningfully modify the AD trajectory. Early vaccines such as AN1792 demonstrated proof‐of‐concept but were limited by T‐cell‐mediated adverse events. Second‐generation approaches, including CAD106, UB‐311, ABvac40 and ACI‐24, improved safety and immunogenicity through short peptide fragments and liposomal formulations. Next‐generation platforms such as MultiTEP and SupraAntigen, alongside nucleic acid‐based vaccines, further optimise immune responses, overcoming immunosenescence and selectively targeting toxic oligomers while sparing physiological proteins. Tau‐targeted vaccines, including AADvac1, ACI‐35 and AV‐1980R, show selective reduction of pathological Tau with promising preclinical and early clinical results. Combined Aβ/Tau vaccination demonstrates synergistic effects in preclinical models, supporting multi‐target strategies. Future directions focus on preventive vaccination, alternative delivery routes and biomarker‐driven personalisation, collectively supporting a shift towards integrated multi‐target immunotherapy capable of modulating AD pathology and potentially altering its long‐term clinical course.

## Graphical abstract

Overview of immunotherapeutic strategies targeting Alzheimer's disease (AD) pathology and their clinical implications. (Left) Key neuropathological hallmarks of AD, including extracellular amyloid‐beta (Aβ) aggregates and intraneuronal hyperphosphorylated Tau (phospho‐Tau), converge to produce synaptic failure, neuroinflammation and neuronal degeneration. (Centre) Active immunisation strategies are classified by generation: early vaccines (AN1792) provided proof‐of‐concept but were associated with T‐cell‐mediated adverse events; second‐generation vaccines (CAD106, UB‐311, ABvac40 and ACI‐24) employ short peptides and liposomal formulations to improve safety; next‐generation platforms (MultiTEP and SupraAntigen) leverage nucleic acid and platform‐based technologies for enhanced targeting; Tau‐targeted vaccines (AADvac1, ACI‐35 and AV‐1980R) achieve selective Tau immunisation; and combined Aβ/Tau approaches pursue synergistic multi‐target effects. (Right) Future clinical directions include preventive immunisation, alternative delivery routes and biomarker‐driven personalisation, collectively converging towards a multi‐target immunotherapy paradigm capable of modifying the AD disease trajectory.

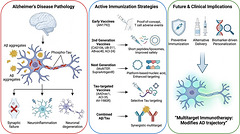

## 1 Introduction

Alzheimer's disease (AD) is the leading cause of dementia, accounting for approximately 60%–70% of all cases worldwide [1]. It currently affects millions of individuals and poses an escalating public health and socioeconomic challenge. In 2025, more than 7 million Americans live with AD, with numbers expected to nearly double by 2050 [2]. In Europe, approximately 9 million people are affected, with the prevalence predicted to exceed 14 million by 2050 [3]. Globally, the economic burden of dementia has already surpassed $1.3 trillion annually and is expected to double within two decades [4, 5]. The impact of AD, however, is unevenly distributed. Recent analyses have shown marked disparities in care costs across Europe, ranging from €8000 in Eastern countries to over €70,000 in the British Isles, and similar state‐ and ethnicity‐based differences in the United States, particularly affecting African American and Hispanic populations [6, 7]. These inequalities, compounded by limited access to specialised care and persistent stigma, further amplify the global human and societal burden of the disease [8].

Pathologically, AD is characterised by the abnormal aggregation of extracellular β‐amyloid (Aβ) and intracellular hyperphosphorylated Tau, leading to synaptic dysfunction, mitochondrial impairment, neuroinflammation and progressive neuronal loss [9, 10, 11]. Current therapies remain mainly symptomatic, offering only modest and transient cognitive benefits [12, 13]. Recognising AD as a multifactorial proteinopathy has shifted therapeutic strategies towards immunotherapy, aiming to neutralise or prevent the accumulation of toxic Aβ and Tau species before irreversible neurodegeneration occurs [14].

Within this framework, active immunisation represents a promising approach to induce adaptive, long‐lasting immune responses against pathological Aβ and Tau. By promoting endogenous antibody production and T‐cell cooperation, vaccination may prevent the formation of neurotoxic aggregates and mitigate downstream neurodegenerative processes [15, 16]. This review outlines the evolution of active immunisation strategies for AD, from early Aβ‐ and Tau‐based vaccines to next‐generation multi‐target platforms, highlighting key lessons from past trials, advances in antigen design and current challenges in developing truly preventive and disease‐modifying vaccines.

## 2 Vaccination Against Aβ in AD

### 2.1 Rationale for Aβ as a Therapeutic Target

Among the molecular hallmarks of AD, Aβ deposition has long been regarded as a central pathogenic driver. The amyloid cascade hypothesis, first articulated in the 1990s, proposes that the accumulation of Aβ peptides, particularly Aβ_42_, triggers a cascade of downstream events, including Tau hyperphosphorylation, synaptic dysfunction, neuroinflammation and ultimately neuronal loss [10].

The amyloid cascade hypothesis has faced criticism because a substantial proportion of cognitively normal older adults harbour amyloid burdens on PET or in CSF that are indistinguishable from those of symptomatic patients, prompting frameworks that now define AD biologically rather than purely clinically [17], while cross‐sectional Tau‐PET studies show that the regional distribution of Tau tracks with the specific clinical phenotype and pattern of neurodegeneration considerably more closely than amyloid burden does [18]. Although the clinical failures of conventional anti‐Aβ therapies, the weak correlation between plaque burden and cognitive decline, and the recognition that Tau pathology and neuroinflammation are more closely associated with clinical progression have cast doubt on its centrality, converging genetic, biochemical and neuropathological evidence nonetheless supports its pivotal role in the earliest stages of the disease. Indeed, (i) autosomal‐dominant AD mutations consistently increase Aβ production or aggregation [15], (ii) amyloid plaques accumulate years before the onset of clinical symptoms [19] and (iii) soluble Aβ oligomers exert potent synaptotoxic and neurotoxic effects [16]. Autosomal‐dominant mutations, however, account for only a small minority of AD cases overall [20], and it remains an open question how far the mechanisms established in these familial forms generalise to sporadic, late‐onset AD, where genetic risk is instead concentrated in microglial and innate‐immune genes (Section 4). The answer has direct implications for how broadly Aβ‐targeted active immunisation should be deployed.

The rationale for Aβ‐targeted vaccination lies in its potential to mobilise the immune system to recognise and eliminate pathogenic Aβ species. Unlike small‐molecule therapeutics, which must cross the blood‒brain barrier and directly interfere with peptide aggregation, active immunisation can induce durable systemic and central immune surveillance, enabling antibody‐mediated neutralisation of soluble oligomers and promoting microglial clearance of deposited plaques [21, 22, 23]. Importantly, such an approach could also be applied prophylactically in individuals at elevated genetic or biological risk for AD, such as carriers of autosomal‐dominant mutations (APP, PSEN1, PSEN2), APOE ε4 allele carriers, individuals with Down syndrome, or cognitively unimpaired subjects with preclinical amyloid pathology, with the ultimate goal of preventing disease onset rather than merely mitigating its symptoms [16, 19].

### 2.2 Preclinical Studies—Active Immunisation in Transgenic Models

The first demonstrations of Aβ vaccination efficacy originated from transgenic mouse models of AD. In 1999, Schenk et al. reported that immunisation of PDAPP mice, which overexpress human APP carrying the V717F mutation, with full‐length human Aβ_42_ peptide emulsified in Freund's adjuvant elicited high‐titre anti‐Aβ antibodies. This immune response led to a marked reduction in amyloid plaque burden, decreased neuritic dystrophy, and improved memory performance in behavioural tasks [24]. This pivotal study provides the first proof‐of‐concept that active immunisation can simultaneously ameliorate neuropathological features and cognitive deficits associated with Aβ accumulation.

Building on this work, Bard et al. demonstrated that passive transfer of monoclonal anti‐Aβ antibodies into PDAPP mice could also reduce amyloid deposition, establishing that antibody‐mediated clearance alone was sufficient to achieve therapeutic effects even in the absence of T‐cell activation [21]. This observation was crucial, as it suggested that antibodies themselves, rather than cytotoxic T‐cell responses, were the main drivers of Aβ removal, laying the groundwork for subsequent passive immunotherapy strategies.

**FIGURE 1:**
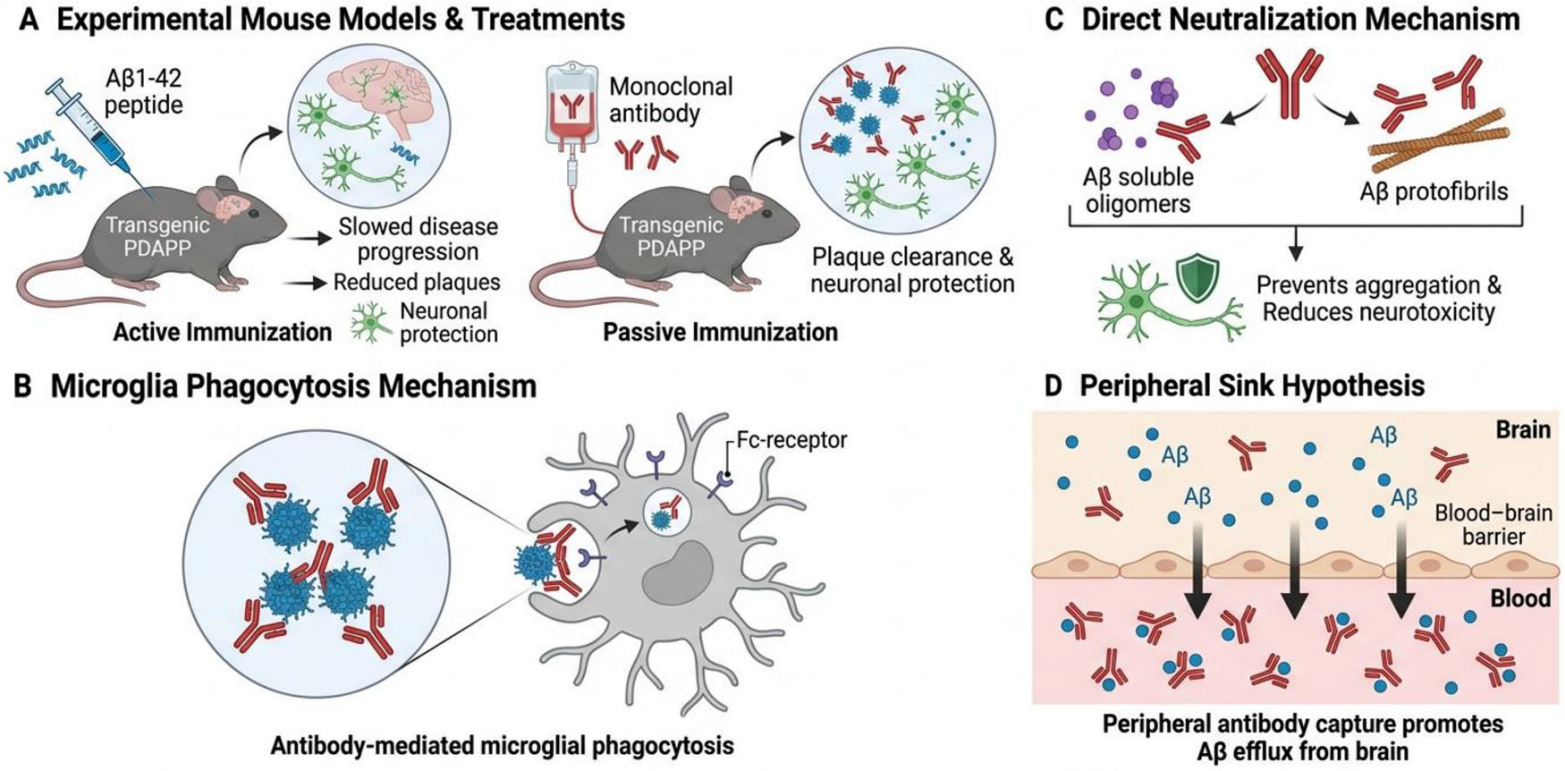
Preclinical studies of active and passive Aβ immunotherapy in amyloid mouse models. The initial proofs‐of‐concept for active and passive immunotherapy targeting the Aβ_1‐42_ peptide in Alzheimer's disease and the hypothetical clearance mechanisms. (A) Schenk et al. demonstrated that in PDAPP transgenic mice, active immunisation with Aβ peptides (left) significantly reduced disease severity, slowed progression in older animals, and prevented amyloid plaque formation in younger animals, with no major autoimmune or inflammatory side effects initially observed. Subsequent Bard et al. showed that passive immunisation (right) with monoclonal antibodies against the N‐terminus of Aβ induces plaque clearance and reduces neuropathology and neuronal protection. Panels (B‐D) illustrate the three main mechanisms proposed for clearing Aβ in the brain: (B) microglia phagocytosis, (C) direct action and peripheral sink hypothesis (D). In panel B, antibody (red) acts as a “tag” on the Aβ, making it easier for microglia to identify and remove the plaque via phagocytosis‐Fc‐mediated; in panel (C), antibody binds directly to Aβ soluble oligomers (purple) and protofibrils (brown), preventing their further aggregation and thereby neutralising their neurotoxic effects; in panel (D), in the blood compartment, circulating antibodies capture soluble Aβ, lowering peripheral levels and promoting the efflux of brain‐derived Aβ across the blood–brain barrier (light brown), enhancing overall clearance. The figure was created with Biorender.com.

Proposed mechanisms of Aβ clearance include several complementary processes: (i) opsonisation and microglial phagocytosis, in which antibody‐tagged amyloid aggregates are recognised and engulfed by microglia, leading to plaque degradation [22], (ii) peripheral sequestration of soluble Aβ, the so‐called ‘peripheral sink’ effect, whereby antibodies in the bloodstream bind circulating Aβ, shifting the equilibrium and promoting efflux of Aβ from the brain [25] and (iii) neutralisation of soluble toxic oligomers, preventing their synaptotoxic and neuroinflammatory actions [23]. Figure 1 summarises the proof‐of‐concept for Aβ immunotherapy, highlighting the key findings from 1999 and 2003 and the proposed mechanisms for Aβ clearance, including microglial phagocytosis, direct action and the peripheral sink hypothesis.

Importantly, no major autoimmune or inflammatory side effects were observed in these preclinical models [26, 27], reinforcing confidence in the safety and feasibility of immunotherapy and paving the way for translation to human clinical trials.

Overall, these preclinical studies not only validated Aβ as a therapeutic target but also established experimental paradigms, both active and passive immunisation, that dominated AD immunotherapy research for the following two decades. By the late 1990s, enthusiasm for clinical translation was high, and the first human trials were soon underway.

### 2.3 The First Human Trial: AN1792—Successes and Failures

The first human trial of active Aβ immunisation, AN1792 (Elan Pharmaceuticals, 2000), employed the full‐length Aβ_42_ peptide formulated with the QS‐21 adjuvant. Early Phase I results demonstrated that patients with mild‐to‐moderate AD were able to mount a specific antibody response against Aβ, confirming that active immunisation was both feasible and immunogenic in humans [28].

Encouraged by these findings, a larger multicentre Phase IIa trial was launched to further evaluate safety and potential efficacy. However, the study was prematurely discontinued in 2002 after a subset of participants developed meningoencephalitis, attributed to a T‐cell‐mediated autoimmune response against Aβ [29]. Although the incidence of this adverse effect was relatively low, its severity underscored the risk of using full‐length self‐antigens capable of eliciting cytotoxic T‐cell activation.

Neuropathological analyses of vaccinated individuals provided crucial insights. Both Nicoll et al. and Ferrer et al. reported extensive cortical amyloid clearance in some regions, nearly complete, accompanied by microglial activation and phagocytic activity, demonstrating that antibody‐mediated plaque removal can indeed occur in the human brain [30, 31]. Nonetheless, neurofibrillary tangles (NFTs), neuronal loss, and gliosis persisted, and in some cases, perivascular T‐cell infiltrates were observed, indicating ongoing inflammatory responses despite plaque removal.

Clinically, cognitive outcomes were modest: vaccinated patients generally continued to decline, suggesting that Aβ removal at symptomatic stages may be insufficient to alter the disease trajectory [32, 33]. Overall, the AN1792 trial highlighted both the promise and limitations of Aβ‐targeted vaccination, demonstrating that while the immune system can be effectively mobilised against its pathological target, translating amyloid clearance into meaningful clinical benefit remains a major challenge, and one that subsequent active immunisation strategies have sought to address, as detailed in Tables 1 and 2, which overview the active anti‐Aβ vaccines under development and trace the clinical history of each candidate, respectively.

**TABLE 1:** Molecular strategy, platforms and specificity of active immunisation vaccines against Aβ.

Generation	Vaccine	Company	Epitope	Adjuvant	Platform	Composition	Specificity for pathological species	Mechanism of Action
Early^a^	AN1792	Elan/Wyeth	Full‐length Aβ_1–42_	QS‐21	Peptide	Pre‐aggregated Aβ_1–42_	Fibrillar/insoluble Aβ (plaques)	Active immunisation; induces antibodies against plaques; triggers Th1‐mediated response: clearance of deposits
Second^b^	CAD106	Novartis Pharmaceuticals	N‐terminal Aβ_1–6_	Alum	Peptide–carrier conjugate	Aβ_1–6_ linked to Qβ bacteriophage	Soluble (oligomers, monomers) and aggregated (fibrils, plaques) Aβ	Induces a Th2‐biased immune response, promoting antibody‐mediated clearance of toxic Aβ species
Second	ACC‐001	Pfizer, Janssen	N‐terminal Aβ_1–7_	QS‐21	Peptide–carrier conjugate	Aβ_1–7_ linked to KLH coupled to inactivated diphtheria toxin	Soluble (monomers, oligomers) and aggregated (fibrils, plaques) Aβ	Antibody‐mediated clearance; Th2‐biased response
Second	ACI‐240.60	AC Immune, SA, Takeda	N‐terminal β‐sheet stabilised Aβ_1–15_	MPLA	SupraAntigen	Liposomal Aβ_1–15_ β‐sheet	Oligomers; pyroglutamate‐Aβ_3–42_	Induces antibodies targeting β‐sheet structure; promotes microglial clearance; reduces toxic oligomers; Th2‐biased response
Second	UB‐311^c^	Vaxxinity	N‐terminal Aβ_1–14_	CpG + Alum	UBITh	Mix of synthetic Aβ_1–14_ peptides linked to MVF_288–302_ and synthetic Aβ_1–14_ peptides linked to HBsAg_19–33_	Fibrils; oligomers	Th2‐biased antibody response; promotes clearance of Aβ oligomers and plaques
Second/emerging^d^	ABvac40	Axon Neuroscience SE	C‐terminal Aβ_34–40_	Alum	Peptide–carrier	Aβ_33–40_ conjugated to KLH	Oligomers and fibrils	Th2‐biased antibodies; recognition of toxic Aβ_40_; microglia clearance; reduction of plaques/oligomers
Emerging^e^	ALZ‐101	Alzinova AB	Stabilised Aβ_42_ oligomer	Aluminium hydroxide	Peptide‐based vaccine	AβCC peptide	Selectively recognises neurotoxic Aβ soluble oligomers, minimising binding to monomeric or fibrillar forms	Induces antibodies that selectively neutralise soluble Aβ oligomers, reducing synaptotoxicity and amyloid pathology
Emerging	AV‐1959D	AC Immune	Aβ_1–11_	—	MultiTEP	DNA vaccine encoding N‐terminal Aβ	Soluble (monomers, oligomers) and aggregated (fibrils, plaques) Aβ	Self‐adjuvant; Th2‐biased antibody response
Emerging	AV‐1959LR	AC Immune	Aβ_1–11_	—	MultiTEP	mRNA vaccine encoding N‐terminal Aβ	Soluble and aggregated Aβ species	Expressed in situ; induced antibody response in vivo; flexible platform for multi‐epitope design
Emerging	AV‐1959R	AC Immune	Aβ_1–11_	—	MultiTEP	Recombinant protein	Soluble and insoluble Aβ; may cross‐react with Tau epitopes	Multi‐epitope induction; Th2‐biased antibody response; flexible for combinatorial targets

*Note*: Conceptual overview and classification of active Aβ vaccines, grouped by generation (early, second, second‐emerging and emerging). The table indicates the molecular strategy of each candidate. Key parameters include the targeted epitope (e.g., N‐terminal or C‐terminal), the adjuvant, the platform used (e.g., peptide–carrier, DNA‐MultiTEP, mRNA) and their specificity for pathological species (e.g., fibrils, oligomers or selectivity for neurotoxic oligomers such as ALZ‐101). Finally, it summarises the mechanism of action, highlighting the shift from a T‐cell‐mediated response to a Th2‐biased response, focused on antibody‐mediated clearance.

^a^ First demonstration that active anti‐Aβ immunisation can reduce plaques; failed due to excessive T‐cell response.

^b^ Focusing on the N‐terminal fragment and non‐autoreactive carriers: Th2‐biased response, improved safety.

^c^ UB‐311 contains a mixture of two different Aβ_1–14_ peptide antigens linked to different T‐helper epitope.

^d^ Focused on the C‐terminal fragment of Aβ_40_ with a non‐autoreactive carrier: Th2‐biased response, improved safety; highly selective design, potentially combinable with other therapies.

^e^ Genetic platforms (DNA/mRNA/MultiTEP): flexible immunisation, combinable with multiple targets (Aβ + Tau).

**TABLE 2:** Detailed clinical chronology and current status of active immunisation vaccine candidates against Aβ.

Vaccine	Trial ID (NCT)	Phase—Start and end	Population	Dosing and regimen	Main outcomes	Status/notes
AN1792	—	I from Apr 2000 to Jun 2002	Mild–moderate AD, multicentre (enroled: 64)	50 or 225 µg, IM	Safe, immunogenic, transition to Phase II	Completed
AN1792	NCT00021723	IIa from Sep 2001 to Sep 2003	Mild–moderate AD, multicentre (*n* = 299, enroled: 375)	50 µg up 12 injections (estimated), IM	6% encephalitis; reduced amyloid plaques post‐mortem; no cognitive benefit	Terminated (safety concerns)
ACC‐001	NCT00479557 (UE) and NCT00498602 (US)	IIa from May 2007 to Jan 2013	Mild–moderate AD, age 50–85 years (enroled: 245), 18 months	3, 10 or 30 µg ± QS‐21 adjuvant, five injections, IM	Safe, immunogenic, slightly decreased p‐Tau in CSF No cognitive benefit; local injection reaction and headaches ARIA‐E occurs in few cases	Completed
ACC‐001	NCT00955409 (extension UE), NCT00960531 (extension US)	IIa from Giu 2009 to Jul 2013	Mild–moderate AD, age 50–85 years (enroled: 160), 104 weeks	3, 10 or 30 µg ± QS‐21 adjuvant, IM	Safe, robust antibody response, well‐tolerated ARIA‐E occurs in few cases (in ≥ 5% of total subjects). No cognitive benefits	Completed, terminated (lack of clinical effect)
ACC‐001	NCT01238991 (extension Japanese)	IIa from Dec 2010 to Jul 2013	Mild–moderate AD, age 52–87 years (*n* = 21, enroled: 21), 104 weeks	3, 10 or 30 µg, IM	Safety and well‐tolerated, QS‐21 increases antibody response ARIA‐E occurs in few cases (in ≥ 2% of total subjects). No cognitive benefits	Terminated (lack of clinical effect)
ACC‐001	NCT00752232 (Japanese)	II from Dec 2008 to Jul 2012	Mild to moderate AD, age 50–85 years (*n* = 16, enroled: 16), 104 weeks	3, 10 or 30 µg, IM	Safety and immunogenic; well‐tolerated. AEs in injection sites. No cognitive benefits No ARIA	Completed
ACC‐001	NCT00959192 (Japanese)	II from Aug 2009 to Jan 2013	Mild–moderate AD, age 50–85 years (*n* = 54, enroled: 65), 104 weeks	3, 10 or 30 µg, IM	Safety and well‐tolerated, QS‐21 increases antibody response No cognitive effect No ARIA	Completed
ACC‐001	NCT01284387	II from Jan 2011 to Feb 2014	Mild–moderate AD, age 50–85 years (*n* = 86, enroled: 126), 104 weeks	3 or 10 µg, IM	Safe and well tolerated; strong immune response; no significant cognitive or biomarkers improvement	Completed
ACC‐001	NCT01227564	II from Feb 2011 to Feb 2014	Early AD, age 50–80 years (*n* = 42, enroled: 63), 104 weeks	3 or 10 µg, IM	Safe, immunogenic; no clear clinical benefit	Completed
ACC‐001	NCT00955409 (long‐term extension)	II from Nov 2009 to Dec 2013	Mild–moderate AD, age 50–85 years (*n* = 159, enroled: 160), 104 weeks	3, 10 or 30 µg, IM	Safety, tolerability profile and robust antibody responses; no cognitive improvement or amyloid reduction	Completed, terminated (lack of clinical effect)
CAD106	NCT00411580	I from Aug 2005 to Mar 2008	Mild–moderate AD	50–450 µg, IM	Well‐tolerated and strong antibody response; no cognitive benefit	Completed
CAD106	NCT00733863 (2201, Europe); NCT00795418 (2202, US); NCT00956410 (Extension 2201); NCT01023685 (Extension 2202)	IIa Study 2201, from 2008 to Feb 2010, Study 2202, from Oct to Nov 2008; Extension_2201E, from Sep 2009 to Jul 2011; Extension_2202E, from Dec 2009 to Feb 2012	Mild AD age 40–85 years, 52 weeks; age 40–85 years, 52 weeks; age 40–85 years, 66 weeks; age 40–85 years, 66 weeks	150 µg, IM, three doses; 150 µg, SC/IM, three doses; 150 µg, SC, four doses; 150 µg, SC, four doses	In all trial, confirmed safety and tolerability; no cognitive improvement	Completed
CAD106	NCT01097096	IIb from Mar 2010 to Mar 2011	Mild AD, aged < 85 years (*n* = 106, enroled: 121), 90 weeks	150 or 450 µg seven dosages, IM	82% responders; acceptable safety and tolerability; biological signals of amyloid reduction. AEs: headache, hypertension, and pyrexia. No cognitive improvement	Completed
CAD106	NCT02565511	II/III from Mar 2006 to Sep 2019	At risk for AD (APOE ε4 homozygotes), age 60–75 years (*n* = 42, enroled: 480), 260 weeks	450 µg four doses, IM	PET amyloid reduction trend; AEs: headache, local injection	Terminated (negative results from CNP520; sample size very small)
UB‐311	NCT00965588	I from Feb 2009 to Apr 2011	Mild–moderate AD, age 50–85 years (enroled: 19), 24 weeks	300 µg three doses, IM	100% responders; adverse event in site injection and agitation	Completed
UB‐311	NCT02551809	IIa from Dec 2015 to Aug 2018	Mild AD, age 60–90 years, 78 weeks (*n* = 30 with 22 patients APOE4 carriers, enroled:45)	300 µg, IM	97% responders; safety, well‐tolerated, immunogenicity; no adverse events, clinical outcomes were promising but no statistically significant (small sample size)	Completed
UB‐311	NCT03531710	IIa from Aug 2018 to Oct 2019	Mild AD, age 57–87 years (enroled: 34 estimated), 108 weeks (estimated)	300 µg, three or five doses, IM	97% antibody responders; well‐tolerated; dementia severity, cognitive performance, progression disease (PET, CDR‐SB, MMSE) directional trend favouring (no statistically significant, small size sample)	Terminated (treatment assignment error)^a^
ACI‐24	NCT02738450	Ib/II from Apr 2016 to Jul 2018	Adults with Down syndrome at risk AD, age 25–45 years, (*n* = 12, enroled: 16), 96 weeks	300 or 1000 µg seven doses, SC	Safe, well‐tolerated, mild local reactions, modest antibody response; decrease insoluble Aβ_40/42_ and soluble Aβ_42_ No significant adverse effects	Completed
ACI‐24	NCT05462106	Ib/II (ACI‐24.060) From Jun 2020 to Jun 2026, estimated	Prodromal AD, age 50–85 years; adults with Down syndrome at risk, age 35–50 years (enroled: 140 estimated)	Different dosages to establish	Well‐tolerated, no serious adverse events; higher immunogenicity; PET and cognition outcomes ongoing	Recruiting/ongoing
ABvac40	NCT03113812	I from Dec 2013 to Mar 2015 (with a temporary suspension from Jul 2014 to Jan 2015 for interim analysis and protocol amendment)	Mild–moderate AD, age 50–85 years (*n* = 16, enroled: 24), 16 weeks	100 or 200 µg, SC, two or three doses, respectively	Good antibody response (∼92% of responders in group with three injections) safety and tolerability	Completed
ABvac40	NCT03461276	II from Dec 2017 to Jul 2021	a‐MCI or Vm‐AD, age 55–80 years (*n* = 66, enroled: 238), 36 months	200 µg, five monthly injections plus a 10‐month booster	Good safety and well‐tolerated; strong antibody response; reduced the risk of clinically meaningful cognitive decline; MMSE, CDR‐SB, and ADAS‐Cog were all directionally favourable. No ARIA‐A or ARIA‐E	Completed transition to Phase III
AV‐1959D	NCT05642429	I from Feb 2023 to Feb 2026, estimated	Patients with early Alzheimer's disease (≈48 participants)	Three dose cohorts vs. placebo, ID	Safety and tolerability primary; immunogenicity expected (preclinical strong)	Active, not yet fully recruited/completed (as of available info)
AV‐1959LR	Preclinical pilot stage	Preclinical/early human planning	Animal models (mice, NHP)	mRNA in LNP (dose escalation in animals)	High anti‐Aβ antibody titres in mice/NHP; immunogenicity shown	Human trial not yet reported or early stage
AV‐1959R	Phase I results announced	I	Healthy volunteers/primary and secondary prevention risk groups	Adjuvanted recombinant MultiTEP Aβ epitope vaccine	Safe, immunogenic in Phase I (press release)	Further Phase II design ongoing (for prevention)

*Note*: Clinical record tracing the developmental history of active anti‐Aβ vaccines through human clinical trials. For each study, it documents the Trial ID (NCT), the development phase, and logistical details, such as the population enroled (e.g., mild‐to‐moderate AD or at‐risk AD) and the specific dosing regimen. The main outcomes section reports key results on safety, tolerability and immunogenicity, along with cognitive and biomarker efficacy data. The status/notes column is crucial for understanding the clinical chronology, documenting terminations due to safety concerns (e.g., AN1792) or lack of clinical benefit (e.g., ACC‐001 and CAD106) and the current status of ongoing trials (such as ACI‐24).

^a^ According to Triplett et al., a Phase III development plan for UB‐311 was presented at the CTAD 2020 conference, involving two identical randomised, double‐blind, placebo‐controlled trials (∼3200 participants with MCI or early AD). The FDA granted the Fast Track designation in May 2022, but no updates on trial initiation have been reported as of 2023 [116].

### 2.4 Second‐Generation Active Vaccines

Second‐generation anti‐Aβ vaccines were developed following the safety concerns raised by AN1792, which demonstrated risks of T‐cell‐mediated neuroinflammation associated with full‐length Aβ peptides and strong adjuvants [29, 30]. These newer formulations employ shorter, non‐self‐reactive Aβ fragments, mainly from the N‐terminal region, and platforms with intrinsic adjuvanticity to elicit a strong humoral response while minimising T‐cell activation and CNS inflammation [33, 34]. The resulting polyclonal antibodies can target multiple Aβ species, including soluble oligomers and fibrillar aggregates, thereby facilitating amyloid clearance and limiting further deposition with improved safety [35]. Overall, these second‐generation vaccines, reviewed below, mark a safer and more refined approach to active immunotherapy for AD.

*CAD106* emerged as the leading candidate in this class. It incorporates the Aβ_1–6_ fragment, chemically coupled to a bacteriophage Qβ virus‐like particle (VLP). The VLP's highly repetitive capsid structure provides intrinsic adjuvant activity, promoting immune recognition and innate activation through toll‐like receptor signalling. CAD106 specifically targets monomeric Aβ and induces a strong humoral response while avoiding Aβ‐specific T‐cell activation. Nevertheless, the induced antibodies can cross‐react with aggregated Aβ species, including oligomeric and fibrillar forms [36]. Preclinical studies demonstrated robust antibody induction and plaque reduction without T‐cell‐mediated inflammation, supporting advancement to multiple Phase I [37] through Phase II/III clinical trials [38]. In clinical trials, CAD106 consistently induced strong and sustained anti‐Aβ antibody responses with a favourable safety profile, showing no encephalitic events like those seen with AN1792. Long‐term follow‐up confirmed good tolerability after repeated dosing, although cognitive improvements were limited. In a Phase IIB study, CAD106 treatment led to a measurable reduction in amyloid plaques [38], but a subsequent Phase II/III trial comparing CAD106 with a BACE‐1 inhibitor was terminated early due to adverse events in the control group, leading to discontinuation of CAD106 development (NCT0256551).

*ACC‐001* (vanutide cridificar; Janssen/Novartis), a second‐generation conjugate vaccine, consists of a short N‐terminal Aβ peptide (Aβ_1–7_) covalently linked to keyhole limpet haemocyanin (KLH), a non‐self‐carrier protein that provides foreign T‐helper epitopes to promote a robust B‐cell‐mediated antibody response against Aβ. The formulation includes the QS‐21 adjuvant, the same saponin‐based component used in AN1792, but administered in a more controlled and standardised conjugate system to limit systemic inflammation and meningoencephalitic risk while preserving immune potency. The induced antibodies primarily recognise soluble and monomeric Aβ, with partial cross‐reactivity to oligomeric and fibrillar species [39], consistent with modest amyloid reductions observed in imaging and post‐mortem analyses. Clinical studies confirmed robust, sustained antibody titres and favourable tolerability, even after repeated dosing [39, 40, 41]. However, due to limited clinical efficacy, development was discontinued after Phase II [42].

*UB‐311* is a next‐generation synthetic peptide vaccine that employs the UBITh platform, combining short Aβ_1–14_ fragments with non‐self helper T‐cell epitopes to promote a controlled and targeted immune response. In its formulation, two distinct Aβ_1–14_ constructs are used: one fused to a peptide sequence derived from the measles virus fusion protein and the other linked to a fragment of the hepatitis B surface antigen. These conjugates are complexed with CpG oligodeoxynucleotides, forming microparticulate immunostimulatory assemblies that are then adsorbed onto Adju‐Phos, favouring a Th2‐oriented humoral response [43]. Through this configuration, UB‐311 delivers selective T‐cell help to B cells, enhancing antibody production while avoiding autoreactive T‐cell activation and excessive inflammation. The vaccine is designed to recognise pathogenic aggregated Aβ species, including oligomers and insoluble plaques, while sparing monomeric Aβ. Preclinical studies in several animal models demonstrated that UB‐311 induces N‐terminal anti‐Aβ antibodies capable of neutralising toxicity and facilitating plaque clearance without triggering T‐cell‐mediated inflammation [44]. In Phases I and IIa clinical trials, UB‐311 showed an excellent safety profile, high seroconversion rates, and sustained antibody titres, with preliminary signals of cognitive stabilisation in patients with mild AD [45]. Despite limitations such as small sample sizes and occasional protocol deviations, the vaccine has been granted FDA Fast Track designation, and Phase III trials are ongoing to further assess its efficacy and safety [46].

*ABvac40*, developed by Araclon Biotech, is an active vaccine that uses a synthetic peptide corresponding to the C‐terminal region of Aβ_40_ (amino acids 33–40), implicated in amyloid plaque formation in AD, to stimulate a selective humoral immune response. The elicited antibodies predominantly recognise soluble monomeric Aβ_40_, while partially binding oligomers and extracellular fibrils, thereby promoting clearance of soluble peptide and limiting new aggregation without triggering significant inflammatory or autoimmune effects [47]. In Phase I, the vaccine demonstrated favourable safety and induced anti‐Aβ_40_ antibodies in 11 of 12 immunised patients. In the Phase II randomised, double‐blind, placebo‐controlled trial [48], involving approximately 124 patients with amnestic MCI or very mild AD, ABvac40 maintained a comparable safety profile to placebo, with no cases of *a*myloid‐*r*elated *i*maging *a*bnormalities (ARIA) with oedema or effusion (ARIA‐E) or meningoencephalitis and a similar incidence of ARIA with haemosiderin deposition (ARIA‐H, or microhaemorrhage). The vaccine induced a robust and sustained antibody response, with titres persisting for at least 18 months and antibodies detectable in cerebrospinal fluid, consistent with engagement of the CNS compartment.

This finding, however, should be interpreted with some caution. Peripherally administered IgG reaches the CSF mainly through the more permeable blood–CSF barrier at the choroid plexus rather than by crossing the blood‒brain barrier properly, and even under favourable conditions, CSF IgG concentrations normally remain at only 0.1%–0.2% of serum levels [49]. CSF titres are therefore not necessarily representative of antibody concentrations within the brain parenchyma itself or at sites of amyloid deposition, where local microvascular permeability, itself heterogeneous and further altered by amyloid‐related neuroinflammation and cerebral amyloid angiopathy, likely determines effective antibody exposure. Direct quantification of the CSF‐to‐serum antibody ratio, alongside regional PET‐based measures of target engagement, would strengthen the interpretation of ABvac40's CNS immunogenicity data considerably and could serve as a general benchmark for future active immunisation trials in AD.

The vaccine induced a robust and sustained antibody response, with titres persisting for at least 18 months and antibodies detectable in cerebrospinal fluid, confirming target engagement within the CNS. Exploratory cognitive analyses revealed a slower decline in the ABvac40 group: patients exhibited a 53% lower risk of clinically meaningful cognitive worsening compared to placebo (hazard ratio [HR] ≈ 0.47; *p* = 0.012), and the effect was even greater in amyloid‐positive participants (HR ≈ 0.38; *p* = 0.005). MRI volumetric data also suggested reduced cortical and hippocampal atrophy. Collectively, the data indicate that ABvac40 is safe, well‐tolerated and immunogenic, with promising disease‐modifying signals. Its selectivity for soluble Aβ_40_ supports its potential preventive or early‐stage therapeutic role and is suitable for chronic or combination regimens. Based on these Phase II outcomes, Araclon Biotech has announced plans to initiate a Phase III trial in a larger cohort with extended follow‐up, although no Phase III registration is yet available [48].

*ACI‐24* is an active Aβ immunotherapy developed by AC Immune using the proprietary SupraAntigen platform, later refined into a liposomal formulation to enhance antigen stability, safety, and manufacturability. The vaccine presents short N‐terminal Aβ fragments (Aβ_1–15_) anchored to the liposomal membrane via palmitoyl chains, forcing the peptide into a β‐sheet conformation that mimics aggregated Aβ oligomers and protofibrils while sparing soluble monomers. This conformation‐specific design elicits antibodies selectively targeting neurotoxic aggregated species, thereby minimising the risk of meningoencephalitis. Preclinical studies showed that ACI‐24 reduced Aβ_40/42_ plaque load and pyroglutamate‐modified species, improved memory performance, and did not trigger inflammatory reactions. Early clinical trials confirmed a good safety profile but modest antibody titres, while studies in individuals with Down syndrome demonstrated strong immunogenicity and favourable tolerability [50]. Introduced in 2016 as the first Aβ peptide vaccine tested for AD in Down syndrome, ACI‐24 underwent a Phase II clinical program initially expected to conclude in 2024; however, the trial (code: NCT04373616) was withdrawn in 2021 to further optimise the vaccine formulation and study design.

The next‐generation formulation *ACI‐24.060* was developed to enhance conformational epitope presentation and incorporate universal T‐helper epitopes (derived from tetanus toxoid fragments), aimed at boosting immunogenicity in elderly populations. The refined lipid composition further improves antigen stability and surface exposure, resulting in higher antibody titres and broader recognition of toxic Aβ conformers [51]. Currently under evaluation in clinical studies, ACI‐24.060 has demonstrated robust immunogenicity, selective binding to oligomeric and fibrillar Aβ, an excellent safety profile, and encouraging disease‐modifying potential [51, 52].

From an immunological design perspective, the convergence of ACI‐24.060 and ALZ‐101 on strict oligomer and protofibril selectivity, sparing monomeric Aβ, addresses a consideration that goes beyond conventional safety refinement. Soluble Aβ monomers have been proposed to function as an inducible component of CNS innate immune defence. Aβ_42_ displays antimicrobial activity against common microbial pathogens, several of which are capable of causing CNS infection at concentrations comparable to established human antimicrobial peptides [53], and in transgenic mice, Aβ rapidly oligomerises around herpes simplex virus type 1 (HSV‐1) particles, limiting viral neuroinvasion and improving survival [54]. If a fraction of monomeric Aβ does participate in CNS innate immune surveillance, vaccines that induce antibodies against the entire monomeric pool could, in principle, compromise this function. This is a concern of particular relevance in an elderly population already characterised by immunosenescence and an increased susceptibility to reactivation of latent neurotropic herpesviruses such as HSV‐1, whose DNA has been found enriched within amyloid plaques relative to surrounding tissue and whose pharmacological suppression with antiviral therapy has been associated with a substantially reduced incidence of dementia in large pharmacoepidemiological cohorts [55, 56]. Conformationally restricted immunogens such as those used in ACI‐24.060 and ALZ‐101, which present Aβ in preformed oligomeric or β‐sheet conformations not readily adopted by physiological monomers, may therefore preserve this proposed protective function while still selectively targeting pathological aggregates. This is a consideration that could be built into the evaluation of future conformation‐selective candidates, for instance, by testing whether vaccine‐induced antibodies retain reactivity against monomeric Aβ in established antimicrobial activity assays.

### 2.5 Emerging and Next‐Generation Aβ Vaccine Strategies

Despite the progress achieved with second‐generation Aβ vaccines, clinical benefits have remained modest, and immunogenicity is often heterogeneous, particularly in elderly individuals. These limitations, stemming from immune tolerance to the Aβ antigen, have driven the development of a new generation of vaccines designed according to principles of immunological precision and antigen structural optimisation [57]. The SupraAntigen and MultiTEP platforms represent the most advanced approaches: the former enhances the conformational presentation of Aβ epitopes, inducing antibodies selectively targeting toxic oligomeric forms [58, 59], while the latter combines universal T‐helper epitopes with B‐cell‐specific epitopes to strengthen T‐B cooperation and overcome immune tolerance [57, 60]. Overall, these innovations mark a paradigm shift, from the simple removal of amyloid plaques to the targeted neutralisation of neurotoxic Aβ species, offering finer and safer control of the immune response [61].

*AV‐1959D* is a DNA vaccine built on the MultiTEP platform that integrates 12 universal T‐helper epitopes from foreign antigens, such as tetanus toxoid, diphtheria toxin and influenza hemagglutinin, to ensure broad HLA coverage and strong T‐cell help. These epitopes are fused to three tandem copies of the Aβ_1–11_ B‐cell epitope, enabling robust antibody generation while bypassing self‐tolerance to endogenous Aβ. In preclinical models, intramuscular administration followed by electroporation elicited high antibody titres against both monomeric and oligomeric Aβ, preventing plaque accumulation without inducing autoimmune inflammation or other safety concerns [62, 63]. These results supported a Phase I trial in early AD patients (60–85 years), evaluating three intradermal doses (500–2000 µg) with safety and tolerability as primary endpoints; completion is anticipated in 2026 [62].

Building on the DNA‐based AV‐1959D, *AV‐1959R* is a recombinant protein formulation of the MultiTEP vaccine designed to enhance immunogenicity in older adults, prolong antibody responses, and reduce off‐target effects. Unlike AV‐1959D, AV‐1959R is the codon‐optimised construct formulated with the AdvaxCpG adjuvant. In murine and non‐human primate models, AV‐1959R elicited robust antibody and cellular responses against both soluble and oligomeric Aβ, significantly reducing plaque burden without notable adverse events [63]. A Phase I trial in healthy adults (40–60 years) evaluated three intramuscular doses (100 or 300 µg) administered at Weeks 0, 4 and 14, followed by an 8‐week follow‐up. Interim results reported strong safety, no serious adverse events or ARIA, and antibody titres threefold higher than expected [64]. AV‐1959R holds potential for both preventive vaccination in at‐risk, Aβ‐negative individuals and therapeutic use in those with elevated Aβ.

A further development of the DNA‐based vaccine AV‐1959D is *AV‐1959LR*, an mRNA‐based construct designed to enhance production speed, optimise the antigenic sequence, improve delivery efficiency and increase immune activation [65]. Formulated in lipid nanoparticles (LNPs), AV‐1959LR elicited high and sustained antibody titres in preclinical models. Notably, antibodies induced by AV‐1959LR predominantly bound the peripheral halo of Aβ plaques, whereas those generated by AV‐1959D also recognised the dense plaque core, suggesting distinct mechanisms of amyloid modulation. AV‐1959LR currently remains at the preclinical stage.

*ALZ‐101* is a peptide‐based vaccine developed by Alzinova AB (Sweden), designed to selectively target toxic Aβ oligomers. Unlike many second‐ and next‐generation Aβ vaccine candidates, ALZ‐101 presents the full‐length Aβ_42_ fragment in a stabilised oligomeric conformation, built on the AβCC peptide platform. The AβCC design chemically stabilises the Aβ_42_ sequence through two intramolecular disulfide bridges, maintaining a defined oligomeric structure. This allows selective recognition of neurotoxic Aβ oligomers while minimising cross‐reactivity with monomeric or fibrillar forms [66]. Preclinical data showed that immunisation with AβCC‐induced antibodies capable of recognising and neutralising pathogenic Aβ oligomers, thereby reducing toxicity in animal models. In a randomised, placebo‐controlled Phase 1b clinical study, ALZ‐101 was well tolerated and highly immunogenic, with most participants developing oligomer‐specific antibodies and preliminary favourable signals observed in selected biomarkers. Asymptomatic cases of ARIA‐H were reported among treated subjects but without clinical sequelae, and the overall safety profile was considered acceptable based on the data available to date. Following these positive Phase 1b results, Alzinova received Fast Track designation and FDA IND approval to advance ALZ‐101 into a Phase II study, aiming to confirm effects on biomarkers and clinical outcomes in larger cohorts [66].

## 3 Vaccination Against Tau in AD and Tauopathies

### 3.1 Rationale for Tau as a Therapeutic Target

The physiological form of Tau, primarily intracellular, stabilises microtubules and regulates axonal transport, also influencing synaptic plasticity and DNA integrity in dendrites and the nucleus. In Tauopathies, including AD, Tau undergoes hyperphosphorylation and truncation, forming oligomers, aggregated filaments and NFTs. The spread of pathological Tau reflects synaptic loss and neuronal dysfunction in cortical regions critical for memory and cognitive functions, correlating with brain atrophy and cognitive decline [67, 68]. The propagation of pathology is associated with the extracellular form of Tau, which represents a key therapeutic target, as it can be cleared by antibodies, blocking neuronal spread. Active vaccination strategies exploit this property by stimulating the production of antibodies specific to pathological Tau, promoting its clearance and reducing the spread of neuronal damage [69].

Mechanistically, this clearance does not require antibodies to passively diffuse across the neuronal plasma membrane. Instead, antibody‐bound extracellular Tau forms immune complexes recognised by Fc‐gamma receptors expressed on neurons and microglia. Receptor engagement triggers endocytosis of the complex and its delivery to the endolysosomal pathway for degradation, while related intracellular Fc‐receptor systems such as TRIM21 can further target internalised complexes for proteasomal degradation [34, 70]. This receptor‐mediated route offers a coherent explanation for how a comparatively small CNS‐penetrant antibody pool can interrupt the cell‐to‐cell, prion‐like propagation of pathological Tau, intercepting it at the point of interneuronal transfer rather than attempting bulk neutralisation of the much larger intracellular Tau pool.

**FIGURE 2:**
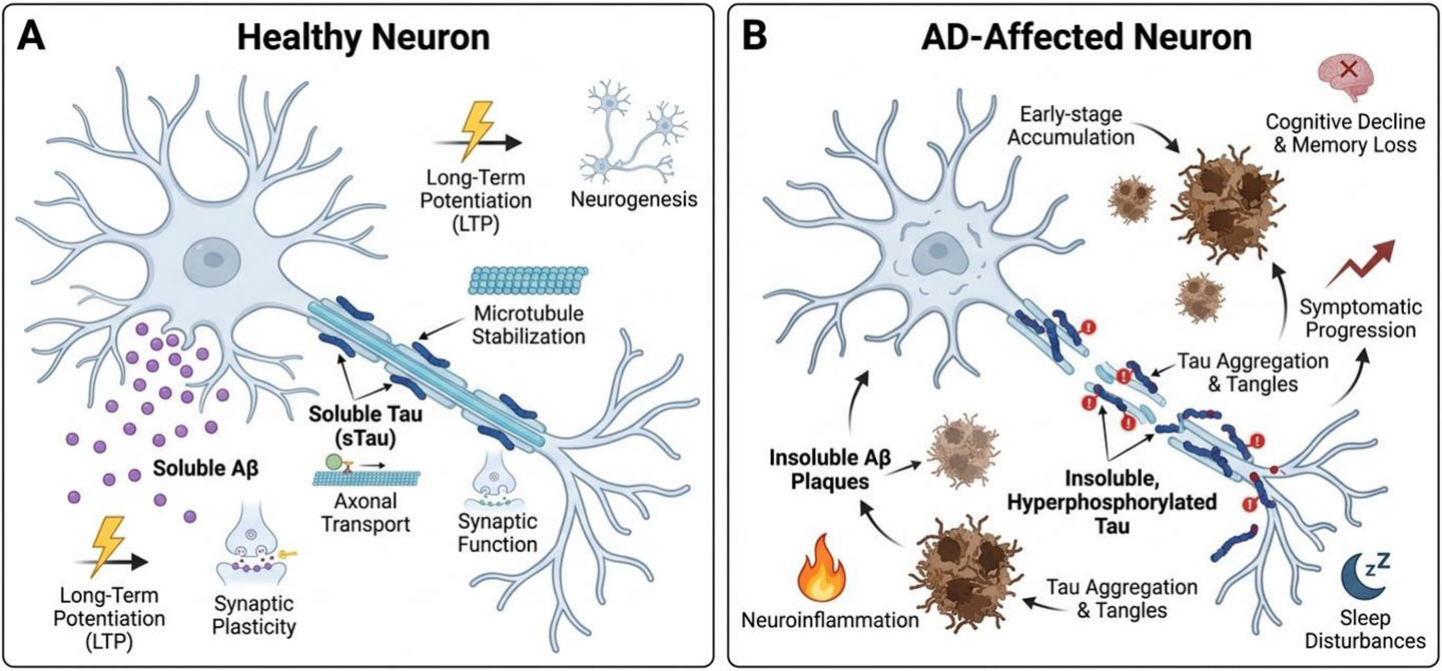
Physiological and Pathological Role of Amyloid‐β (Aβ) and Tau Proteins. The contrast between the function of the soluble and insoluble forms of Aβ and Tau protein in a healthy brain versus a brain with an AD‐dominant phenotype. (A) In a healthy neuron, the soluble forms of Aβ, in purple, and intracellular Tau (sTau), in dark blue, associated with microtubules, in light blue, have beneficial roles, contributing to synaptic plasticity, neurogenesis, long‐term potentiation (LTP), microtubule stabilisation, axonal transport, and synaptic function, respectively. In contrast, in affected neurons (B), the insoluble forms of Aβ, in brown, aggregate into plaques, in dark brown, and insoluble Tau, characterised by hyperphosphorylation, accumulates, leading to widespread neuroinflammation, cognitive decline, and memory loss. Insoluble Aβ is associated with early‐stage accumulation and symptoms, while insoluble Tau is associated with symptomatic progression and sleep disturbances. The figure was created with Biorender.com.

This approach provides a long‐lasting immune response with the potential to modulate neurodegenerative progression, representing a rational complement to anti‐amyloid strategies in AD management [14, 71]. The cellular and regional distribution of Aβ and Tau pathology is not uniform, and this has direct implications for both pathogenesis and antibody‐based clearance. Tau pathology follows a stereotyped topographical progression, beginning in the locus coeruleus and transentorhinal or entorhinal cortex, spreading to the hippocampus and limbic structures and only later involving neocortical association areas [72]. Glutamatergic pyramidal neurons of the hippocampus and cortex, together with noradrenergic neurons of the locus coeruleus, therefore represent the principal cell populations affected by Tau pathology in AD, whereas cholinergic neurons of the basal forebrain are primarily affected as a downstream consequence, underlying the cholinergic deficit targeted by symptomatic therapies [12]. Figure 2 illustrates the physiological versus pathological roles of soluble and insoluble Aβ and Tau in cortical and hippocampal pyramidal neurons, the principal neuronal population relevant to AD vaccination strategies.

### 3.2 Preclinical Immunisation Studies in Tau Transgenic Animals

The first evidence of Tau‐targeted immunotherapy efficacy came in 2007, when Asuni et al. showed that repeated Tau peptide immunisation in P301L transgenic mice, which express mutant human Tau and develop NFTs, reduced pathological Tau and improved cognition without major side effects.

**FIGURE 3:**
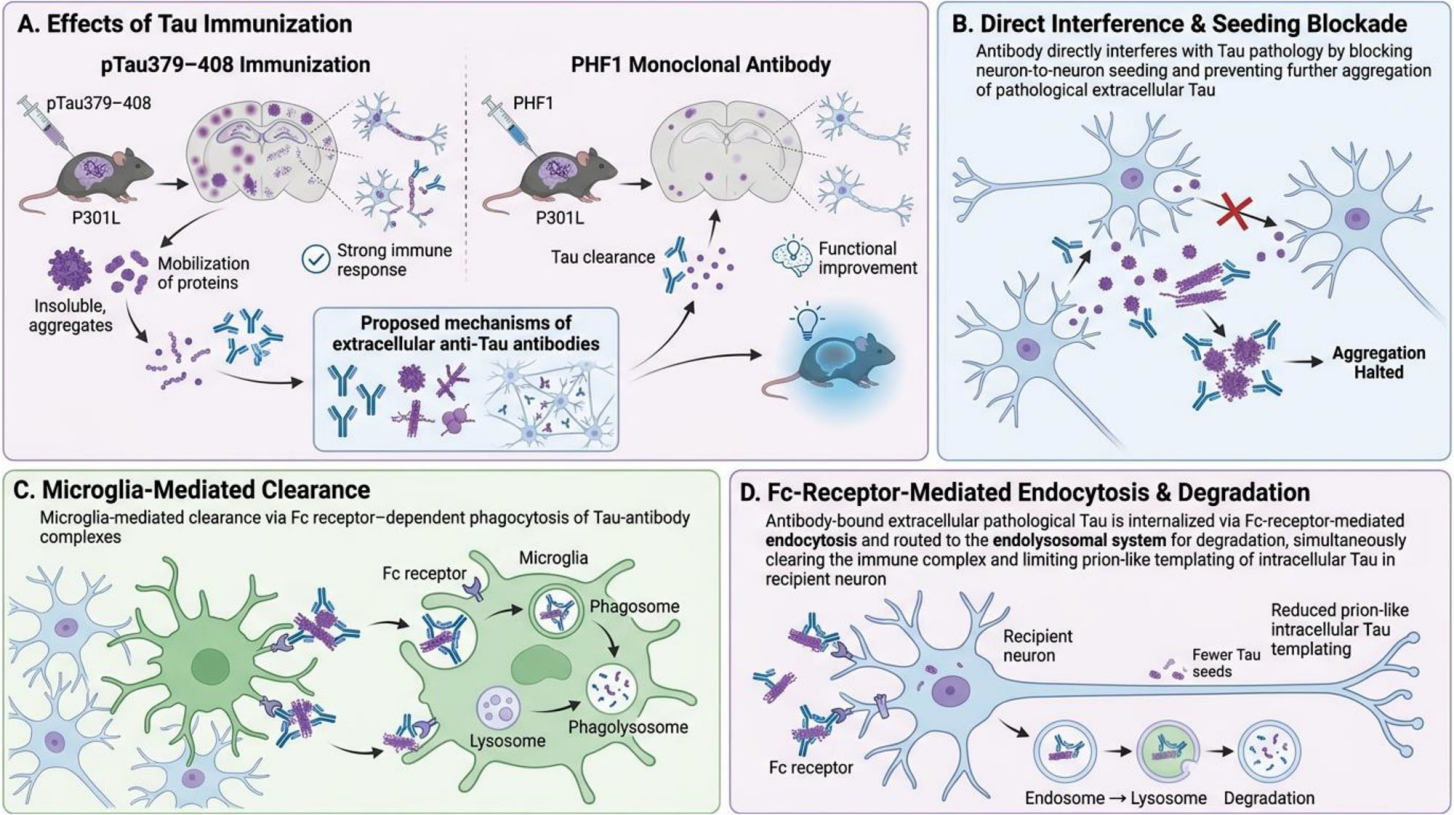
Preclinical studies of active and passive Aβ immunotherapy in P301L mouse models and proposed mechanisms of antibody action. Panel A: (Left) Asuni et al. immunised P301L mice with phosphorylated Tau (pTau379–408), demonstrating a reduction in aggregates in multiple brain regions, mobilisation of protein from insoluble to soluble forms, decreased neuronal Tau pathology, and a strong immune response after repeated monthly injections. (Right) In the same model, Boutajangout et al. administered the monoclonal antibody PHF1 against the C‐terminus of phosphorylated Tau, observing Tau clearance, functional improvement, and reduction in neuronal Tau pathology. (Below) Proposed mechanisms of extracellular anti‐Tau antibodies. Panel B. The antibody directly interferes with Tau pathology by blocking neuron‐to‐neuron seeding and preventing further aggregation of pathological extracellular Tau. Panel C: microglia‐mediated clearance through the Fc receptor–dependent phagocytosis of Tau‐antibody complexes. Panel D: Antibody‐bound extracellular pathological Tau is internalised via Fc‐receptor‐mediated endocytosis and routed to the endolysosomal system for degradation, simultaneously clearing the immune complex and limiting prion‐like templating of intracellular Tau in the recipient neuron.

Later, Boutajangout et al. confirmed similar benefits with passive immunisation using anti‐phospho‐Tau antibodies in JNPL3 mice, another model of Tauopathy carrying the same P301L mutation, promoting extracellular Tau clearance and microglial degradation while preventing neuronal spread [69]. Benefits have also been observed in models of progressive supranuclear palsy (PSP) and corticobasal degeneration (CBD), suggesting broader therapeutic potential beyond AD [71]. These studies established the safety and proof of concept for both active and passive Tau‐targeted therapies in Tauopathies [24, 26, 27]. Figure 3 summarises the proof‐of‐concept for Tau immunotherapy, highlighting the key findings from 2007 and 2011 (Panel A) and the proposed mechanisms for Tau clearance, including blocking neuron‐to‐neuron propagation, microglial phagocytosis and the lysosomal system.

### 3.3 Clinical Vaccine Candidates: Early and Next‐Generation

Encouraging results from anti‐Tau immunisation in transgenic models have paved the way for translation into human clinical studies. Several active vaccines, described below, targeting pathological Tau are currently in clinical development, with AADvac1 and ACI‐35 being among the most advanced.

*AADvac1* is an active anti‐Tau vaccine developed by Axon Neuroscience SE, consisting of a synthetic peptide from the Tau microtubule‐binding domain (aa 294–305: KDNIKHVPGGGS) conjugated to KLH and adjuvanted with alum [73]. The vaccine targets pathological Tau involved in fibrillisation while sparing physiological Tau. In preclinical Tauopathy models, AADvac1 induced strong, selective antibody responses that reduced Tau phosphorylation, oligomer formation, and neurofibrillary degeneration, leading to improved sensorimotor performance and showing a favourable safety profile [73]. The Phase I randomised, placebo‐controlled trial and its open‐label extension confirmed safety, tolerability and high IgG seroconversion in patients with mild‐to‐moderate AD [74]. The FUNDAMANT follow‐up demonstrated sustained antibody titres and associations with slower hippocampal atrophy and cognitive decline [75]. A meta‐analysis further confirmed near‐universal immunogenicity (∼99%) and consistent safety, although without significant clinical benefit [76].

Preliminary results from the Phase II ADAMANT trial indicate reductions in neurodegeneration biomarkers and possible clinical benefit in selected patient subgroups, pending independent confirmation [77, 78]. Overall, AADvac1 exemplifies a first‐generation active Tau immunotherapy, demonstrating the feasibility, safety and biological activity of peptide–carrier–adjuvant vaccine platforms targeting disease‐specific Tau epitopes.

*ACI‐35 (JNJ‐2056)* is an active anti‐Tau immunotherapy originally developed by AC Immune and later licensed to Janssen Pharmaceutica N.V. It uses a liposomal SupraAntigen platform displaying phosphorylated Tau epitopes (pS396/pS404) that mimic the pathological conformation, inducing antibodies selectively targeting aggregated Tau while minimising off‐target immune activation [79]. In preclinical Tauopathy models, ACI‐35 elicited strong anti‐Tau responses, reduced hyperphosphorylated Tau and neurofibrillary pathology, and improved cognition with good tolerability [80]. The Phase 1b trial in mild‐to‐moderate AD confirmed safety but showed limited immunogenicity [79], prompting the development of the optimised ACI‐35.030 formulation. This next‐generation version retained the same pSer396/404 antigen but incorporated enhanced T‐helper epitopes and adjuvants to improve and sustain antibody responses.

A parallel CRM197‐conjugated formulation (JACI‐35.054) and *ACI‐35.030* both showed robust, pTau‐specific responses and cognitive benefits in preclinical models [81]. In the Phase 1b/2a study in early AD, both vaccines were safe and well tolerated, although ACI‐35.030 induced faster, stronger, and more selective antibody responses, supporting its advancement to the Phase 2b ReTain trial in preclinical AD. Now renamed JNJ‐2056, the vaccine received FDA Fast Track designation in 2024 and is being tested in ∼500 Tau‐positive, cognitively normal individuals to evaluate its effects on Tau PET, cognition, and fluid biomarkers [81].

Nucleotide‐ and protein‐based anti‐Tau vaccines developed by the Institute for Molecular Medicine (IMM, CA, USA) represent an innovative strategy for AD prevention and early intervention and are designed to induce selective immune responses against pathological Tau while avoiding autoimmunity. The DNA vaccine *AV‐1980D* encodes three tandem repeats of the Tau_2–18_ sequence from the phosphatase activation domain (PAD), a region normally buried in native Tau but exposed during pathological misfolding. PAD exposure drives Tau aggregation and axonal transport disruption; thus, targeting it enables selective recognition of pathological Tau [82]. Built on the MultiTEP platform, which fuses Tau with universal T‐helper epitopes, AV‐1980D promotes a Th2‐skewed humoral response without self‐reactivity. In THY‐Tau22 mice, intramuscular administration with electroporation induced high‐affinity antibodies recognising extracellular pathological Tau, reducing total and phosphorylated Tau (pS199, AT180) and improving neurobehavioural outcomes. Antibodies also recognise pathological Tau in AD brains, supporting translational potential [83]. These results confirm the preclinical efficacy of the nucleic acid platform, which can elicit both humoral and cellular responses without pro‐inflammatory adjuvants, offering advantages in production flexibility and potential clinical applicability. Currently, AV‐1980D remains in the preclinical stage, with no clinical data available in adults [83].

*AV‐1980R* is a recombinant protein vaccine developed by Michael G. Agadjanyan's group at IMM, California, and belongs to the same series as the Aβ‐targeted AV‐1959R. It uses the MultiTEP platform with universal T‐helper epitopes and contains the Tau_2–18_ B‐cell epitope from AV‐1980D. Switching to a recombinant protein format improved immunogenic potency, predictability and manufacturability, allowing formulation with the AdvaxCpG adjuvant. This configuration elicits durable, Th2‐biased antibody responses, providing strong humoral immunity against pathological Tau without excessive inflammation [83, 84, 85, 86].

In multiple Tau transgenic models (PS19, rTg4510, T5x), AV‐1980R/AdvaxCpG, called AV‐1980R/A, elicited robust anti‐Tau antibodies, reduced total and phosphorylated Tau, and improved cognitive and motor function without T‐cell‐mediated toxicity [84]. Studies in cynomolgus monkeys confirmed strong and durable immunity, the absence of autoreactive T cells, and suitability for cGMP production. Sera from immunised animals bound NFTs and dystrophic neurites in human AD brain tissue. When combined with the amyloid‐targeting vaccine AV‐1959R, AV‐1980R induced parallel, non‐interfering immune responses to both Aβ and Tau, supporting a dual‐target vaccination approach for AD [84]. Whether this preclinical promise translates to clinical benefit will begin to be tested in the upcoming Phase 1 TAURUS‐1980 trial (2025–2029), which will assess AV‐1980R's safety, tolerability and immunogenicity in cognitively unimpaired adults with preclinical AD biomarkers, with doses ranging from 20 to 180 µg and follow‐up to 56 weeks, with the broader landscape of active anti‐Tau vaccination strategies summarised in Table 3 and their clinical histories traced in Table 4.

**TABLE 3:** Molecular strategy, platforms and specificity of active immunisation vaccines against pathological Tau protein.

Generation	Vaccine	Company	Epitope	Adjuvant	Platform	Composition	Specificity for pathological species	Mechanism of Action
Second^a^	AADvac1	Axon Neuroscience SE	Tau aa 294–305 (mid‐domain)	Alum	Peptide–carrier conjugate	Synthetic Tau fragment	Soluble and aggregated phosphorylated Tau (PHFs); mid‐domain epitope	Th2‐biased antibody response; promotes clearance of pathological Tau species
Second	ACI‐35.030	AC Immune SA, Jansenn	Phospho‐Tau pS396/pS404	—	Liposomal peptide	Phosphorylated Tau	Soluble and aggregated phosphorylated Tau; C‐terminal epitopes	Induces antibodies targeting pathological Tau; reduces Tau aggregation and toxicity
Second	JACI‐35.054	Janssen	Phospho‐Tau pS422	—	Liposomal peptide	Phosphorylated Tau	Soluble and aggregated phosphorylated Tau; C‐terminal epitopes	Th2‐biased antibody response; clears phosphorylated Tau aggregates
Emerging^b^	AV‐1980D	AC Immune	Tau_2–18_	—	MultiTEP	DNA encoding N‐terminal Tau	Soluble and aggregated phosphorylated Tau including multiple pathological epitopes	Th2‐biased antibody response; flexible multi‐epitope targeting
Emerging	AV‐1980R	AC Immune	Tau_2–18_	—	MultiTEP	Recombinant protein	Soluble and aggregated phosphorylated Tau including multiple pathological epitopes	Th2‐biased antibody response; designed for combinatorial targeting with Aβ vaccines

*Note*: Conceptual overview and classification of active Tau protein‐targeted vaccines, categorised by generation (second and emerging). The table illustrates the molecular strategy of each candidate, focusing on the pathological epitope targeted, which includes the mid‐domain (AADvac1) or specific C‐terminal phosphorylated epitopes (pS396/pS404 and pS422). The platforms used (e.g., peptide–carrier conjugate, liposome or DNA‐MultiTEP) and the adjuvant are detailed. The specificity for pathological species sections confirms that most vaccines target both soluble and aggregated phosphorylated Tau. The mechanism of action is based on inducing a Th2‐biased antibody response, which is essential for promoting the clearance of pathological Tau species and improving the safety profile.

^a^ Focusing on pathological Tau epitopes (PHF/Tau aggregates, phosphorylated Tau): Th2‐biased response, improved safety; peptide‐ or DNA‐carrier conjugates, with selective targeting of soluble and aggregated Tau species.

^b^ Genetic platforms (DNA/mRNA/MultiTEP): flexible immunisation, combinable with multiple targets (Aβ + Tau).

**TABLE 4:** Detailed clinical chronology and current status of active immunisation vaccine candidates against Tau protein.

Vaccine	Trial (NCT) or preclinical model	Phase—Start and end	Population (treated/total), Age	Dosing, regimen, duration study	Main outcomes	Trial status	References
AADvac1	NCT01850238	I Jun 2013–Mar 2015	Mild‐moderate AD (*n* = 24/30), age 50–85 years	SC, 40 µg, six doses, 48 weeks	Safety and tolerability; Immunogenicity; no significant adverse effects.	Completed	[74]
AADvac1	NCT02031198	Open‐label extension—follow‐up Mar 2014–Aug 2016	Mild‐moderate AD (*n* = 26/NA), age 50–85 years	SC, booster dosages, 72 weeks	Long‐term safety and tolerability Persistence of antibody response	Completed	[75]
AADvac1	NCT02579252 (ADAMANT)	II Jun 2016–May 2017	Mild AD, aged 50–85 years (*n* = 117/79)	40 µg, 11 dosages (booster included), 104 weeks	Safety and tolerability Immuno‐response in > 98% patients AEs in > 80% patients (treated and placebo)	Completed	[78]
ACI‐35.030	NCT04445831	Ib/IIa Jul 2019–Sep 2023	Early mild AD, age 50–75 years (*n* = 26/41)	300, 900 and 1800 µg IM, four doses; follow‐up to Week 74	Good safety and well‐tolerated Rapid (in 96% patients), robust and durable (in 98% patients) polyclonal response in all patients treated A significant (*p* < 0.05) change of plasma p‐Tau and brain‐derived Tau plasma levels; AEs injections site reaction (16.7%–100% patients) and headaches (16.7%–50% patients)	Completed, ongoing Phase 2b, ReTain trial NCT06544616	[81]
ACI‐35.030	NCT06544616 (ReTain)	IIb Sep 2025–Jul 2031 (estimated)	Early AD; enroled ∼500	TBD; multiple IM injections over ∼4 years	Safety; cognition; biomarkers	Ongoing	AC immune announcement
JACI‐35.054	NCT04445831	Ib/IIa Jul 2019–Sep 2023	Early mild AD, age 50–75 years (*n* = 12/16)	15 or 60 µg IM, four doses; follow‐up to Week 74	Good safety and well‐tolerated. Strong but heterogeneous antibody response Required multiple administrations to reach consistent titres	Completed	[81]
AV‐1980D	Preclinical	—	THY‐Tau22 mice	IM + electroporation; multiple doses	Robust anti‐Tau antibody response; reduced total Tau and pS199/AT180 phospho‐Tau	Preclinical	[83]
AV‐1980R/A	Preclinical	—	Not human primate: *Macaca fascicularis*	Multiple injections; Advax‐CpG adjuvant	Strong cellular and humoral responses; antibodies recognise pathological Tau No activation of autoreactive Tau‐specific T helper cells Supports progression to Phase I human trials	Early human trial registered (NCT07158905)	[57]
AV‐1980R/A	NCT07158905	I Dec 2025–Oct 2029	Early AD Age 65–80 years Enroled 48 patients (estimated) Not yet recruiting	20, 60 and 180 µg, IM, at Weeks 0, 4 and 12, and 36 with follow‐up through Week 56	Safety and tolerability, adverse events, labs, ECGs, MRI, and neurological assessments Secondary objectives: anti‐Tau antibody titres. Plasma biomarker and Tau‐PET changes	Ongoing	

*Note*: Clinical record tracing the developmental history of the main active anti‐Tau vaccines through preclinical studies and human clinical trials. For each study, it documents the Trial ID (NCT) or preclinical model, the development phase, the population enroled (e.g., mild‐to‐moderate AD or early AD), and the specific dosing regimens. The main outcomes section reports key results, particularly those related to safety, tolerability and immunogenicity (e.g., the rapid and durable antibody response of ACI‐35.030). The trial status column highlights the status of completion, ongoing studies (such as the ACI‐35.030 ReTain trial) and plans for future trials.

## 4 Lessons Learned From Two Decades of Active Immunotherapy in AD

Over the past two decades, the development of vaccine strategies for AD has undergone a profound transformation, paralleling advances in the understanding of disease pathophysiology and cerebral immune responses. The trajectory from AN1792 to current multi‐target platforms encodes a series of hard‐won lessons on safety, antigen design, adjuvant selection, therapeutic timing and the limits of amyloid‐centric thinking that collectively define the field's current state and its remaining challenges.

### 4.1 The AN1792 Trial: Proof of Concept and Its Price

The first active anti‐Aβ vaccine, AN1792, tested in patients with mild to moderate AD, provided proof of concept that an immune response directed against Aβ could be effectively induced [33]. However, the trial was halted when approximately 6% of participants developed meningoencephalitis, likely mediated by an Aβ‐specific T‐cell response [29]. Post‐mortem analyses showed a significant reduction in amyloid burden but no corresponding cognitive benefit [30, 87], establishing the two cardinal lessons that would define the field thereafter: that autoreactive T‐cell responses must be avoided and that amyloid clearance alone is insufficient to alter the clinical course of the disease. Notably, clonally expanded CD8^+^ T cells with apparent reactivity towards CNS antigens have since been detected in the cerebrospinal fluid of AD patients independent of any vaccination, including at early, pre‐dementia stages [88]. This raises the possibility that AN1792 amplified a pre‐existing CNS‐resident T‐cell compartment rather than generating an entirely novel autoreactive response. Such distinction could usefully inform prevaccination immune profiling in future Aβ‐targeted active immunisation trials.

### 4.2 Redesigning the Immune Response: Epitope Selection and T‐Cell Help

Beyond epitope truncation, the way T‐cell help is provided is perhaps the single most consequential design choice separating second‐generation formulations from AN1792. AN1792's central failure was not merely the use of a full‐length, aggregation‐prone Aβ_1–42_ peptide but the fact that this self‐antigen retains its own T‐cell epitopes. Immunisation with full‐length Aβ under a potent adjuvant therefore risked breaking peripheral tolerance and generating Aβ‐specific T‐cell clones with direct access to their target at high parenchymal concentration. Essentially all subsequent active anti‐Aβ vaccines have addressed this by physically uncoupling the short B‐cell epitope from the source of T‐cell help, which is instead derived from foreign, non‐self sequences.

The strategies adopted differ considerably in their underlying logic and likely robustness in an immunosenescent population. Carrier‐protein conjugation to KLH, as in ACC‐001 and AADvac1, is the most empirical approach, exploiting a large xenoprotein with broad but loosely characterised immunogenicity. VLP display, as in CAD106's Aβ_1–6_–Qβ conjugate, adds repetitive, multivalent antigen presentation that promotes B‐cell receptor cross‐linking independently of T‐cell help, alongside intrinsic toll‐like receptor engagement by the capsid. UB‐311 derives T‐helper epitopes from defined viral antigens—namely, the measles fusion protein and hepatitis B surface antigen—intended to recruit pre‐existing memory responses from childhood exposure or immunisation, in principle favouring memory over naïve T‐cell activation. The MultiTEP platform, used in the AV‐1959 and AV‐1980 series and in Duvax, generalises this logic most systematically, combining 12 universal T‐helper epitopes from tetanus toxoid, diphtheria toxin and influenza haemagglutinin to maximise HLA coverage and exploit pre‐existing memory responses across a genetically diverse elderly population.

The trajectory moving from undefined xenoprotein carriers towards systematically engineered, memory‐recall‐based T‐helper epitope sets represents the field's most coherent response to the lesson of AN1792. Whether the use of carrier sequences, also widely employed in unrelated paediatric and adult conjugate vaccines, could give rise to carrier‐induced epitopic suppression of the anti‐Aβ response in heavily pre‐immunised individuals is, to our knowledge, not yet directly addressed, although AV‐1959R's report of antibody titres threefold higher than expected [64] argues against a major suppressive effect in that particular cohort.

### 4.3 Adjuvant Selection: From Empiricism Towards Mechanistic Rationale

The choice of adjuvant follows a broadly similar evolution. QS‐21, used in both AN1792 and ACC‐001, is a potent saponin‐based adjuvant with documented capacity to promote Th1 and cytotoxic responses. Its retention in ACC‐001 without recurrence of meningoencephalitis suggests that adjuvant potency on its own was not the principal driver of AN1792's adverse events and that epitope content, namely, the presence of self T‐cell epitopes within full‐length Aβ_1–42_, was the more decisive variable. Alum, used in AADvac1 and ABvac40, is a well‐established, Th2‐skewing adjuvant with a long safety record, although its relatively modest potency in older adults may contribute to the heterogeneous, often low‐titre antibody responses observed, which frequently require repeated boosters. CpG oligodeoxynucleotides, used alone or with alum in UB‐311 and combined with delta‐inulin as the AdvaxCpG adjuvant in the MultiTEP‐based candidates, engage toll‐like receptor 9, a pathway that appears comparatively well preserved in the aged innate immune system and may partly explain the unexpectedly robust titres reported for AV‐1959R. MPLA, used in ACI‐24.060, is a toll‐like receptor 4 agonist already validated as part of adjuvant systems in licensed vaccines for older adults, including the recombinant herpes zoster vaccine discussed in Section 5. This convergence raises the testable possibility that TLR4‐directed adjuvants could contribute to neuroimmune benefits beyond their intended effect on anti‐Aβ titres.

### 4.4 The Limits of Amyloid Clearance: Microglial Biology and the Timing Problem

Clinical results from second‐generation vaccines have been encouraging in terms of immunogenicity: CAD106 induced anti‐Aβ_1–6_ antibody responses in more than 80% of patients without central inflammatory events [37], while UB‐311 elicited responses in over 95% of participants and significantly reduced Aβ oligomers in cerebrospinal fluid [44]. Nevertheless, cognitive outcomes have remained modest, highlighting that the amyloid cascade alone does not fully explain AD pathophysiology and that amyloid removal at symptomatic stages may occur too late. A complementary, mechanistically grounded explanation for this dissociation comes from the genetics and biology of microglial activation. Rare TREM2 loss‐of‐function variants confer a two‐ to four‐fold increase in AD risk [89, 90], and TREM2 signalling is required for the formation of a protective microglial barrier around amyloid deposits, with its loss accelerating Tau spread [91]. Single‐cell transcriptomic studies have characterised a disease‐associated microglia (DAM) state that develops in a biphasic manner: an initial TREM2‐independent phase triggered by damage‐associated molecular patterns, followed by a TREM2‐dependent phase of sustained phagocytic and lipid‐handling gene expression [92]. Chronic engagement of this pathway is linked to NLRP3 inflammasome activation and a self‐sustaining IL‐1β‐driven inflammatory state that can persist independently of the amyloid burden that originally triggered it [93].

Active immunisation generates antibodies that opsonise Aβ and may enhance Fc‐receptor‐mediated phagocytosis, and CAD106 indeed demonstrated measurable plaque reduction. Opsonisation acts on the antigen, however, not on the functional state of the microglia that must execute clearance and resolve the ensuing inflammatory response. In the symptomatic populations enroled in the CAD106 and ACC‐001 trials, microglia have likely already progressed through both phases of DAM activation, with an associated neuroinflammatory cascade that, once self‐sustaining, may no longer track tightly with plaque burden. Under this view, the absence of cognitive benefit despite demonstrable amyloid reduction reflects not only timing relative to irreversible neuronal loss but also a more specific uncoupling between the antigen targeted, that is, Aβ, and the cellular effector compartment, that is, microglia, whose functional state determines whether clearance translates into reduced neuroinflammation. This consideration points directly towards combination strategies pairing active immunisation with agents that modulate microglial activation state, for example, TREM2‐agonist antibodies or complement‐pathway modulators, an avenue that, to our knowledge, remains unexplored in the active‐vaccination context.

Recent advances have focused on conformationally selective immunogens designed to elicit antibodies specifically targeting neurotoxic Aβ oligomers rather than monomers or fibrils. Kaplan et al. optimised a vaccine configuration using epitope mapping and computational modelling to identify Aβ conformers most associated with toxicity, achieving selective antibody responses towards oligomeric species without cross‐reactivity to physiological monomers [94]. An interesting exception within the broader landscape is ABvac40, which targets the C‐terminal epitope of Aβ (Aβ_33–40_) and has reported preliminary signals of cognitive stabilisation in patient subgroups alongside improvements in amyloid biomarkers [45, 48]. This distinguishes ABvac40 from other second‐generation vaccines and suggests that epitope selection and refined immunological design may critically influence clinical efficacy, although these findings require confirmation in larger studies.

### 4.5 Passive Immunotherapy as a Benchmark: Lessons From Lecanemab and Donanemab

In parallel, passive immunotherapies using monoclonal antibodies (mAbs) targeting specific Aβ species have offered a more controlled approach to the same biological question. Lecanemab and donanemab, targeting soluble protofibrils and specific Aβ conformations, respectively, have demonstrated significant efficacy in reducing amyloid deposition and slowing cognitive decline in early AD [40, 95], and their regulatory approval represents a landmark achievement in AD immunotherapy [96, 97, 98]. However, mAbs carry important limitations, including amyloid‐related imaging abnormalities (more frequent in APOE ε4 homozygotes), high costs, the need for repeated intravenous infusions and continuous MRI monitoring, all of which limit large‐scale applicability [99, 100, 101]. In this context, active vaccination represents a more sustainable strategy capable of inducing durable, potentially multi‐target immune responses with a positive impact on disease prevalence, healthcare costs and caregiver burden.

### 4.6 The Shift Towards Tau and Multi‐Target Strategies

Attention has therefore progressively shifted from amyloid to Tau protein, which is more directly associated with neuronal damage and clinical progression. Early anti‐Tau vaccines such as AADvac1 have shown strong immunogenicity and a favourable safety profile, inducing IgG1 antibodies in nearly all participants and reducing biomarkers such as plasma p‐Tau217, although without definitive evidence of cognitive improvement [102]. These experiences highlighted the importance of antigen selection, therapeutic timing and the qualitative features of the immune response.

Together, these results have underscored that mere induction of an antibody response is not sufficient: it must be directed against pathogenically relevant epitopes and initiated within an early therapeutic window, when neurodegenerative mechanisms remain potentially reversible. Accumulated evidence has promoted a more integrated view of AD, now recognised as a multifactorial condition in which Aβ and Tau interact synergistically with neuroinflammatory processes, oxidative stress and immunosenescence [103], which is indeed a conceptual shift that has opened the way to combined Aβ/Tau vaccination strategies capable of acting simultaneously on multiple pathological components.

## 5 Limitations, Perspectives and Conclusions

### 5.1 Immunosenescence and the Challenge of Vaccinating an Ageing Population

Despite significant progress, the design of AD vaccines still faces major challenges intrinsic to the target population. Advanced age entails immunosenescence, characterised by reduced T‐ and B‐cell functionality and attenuated antibody responses, which limits vaccine efficacy and complicates the interpretation of immunogenicity data from trials conducted exclusively in older adults [104, 105].

Next‐generation platforms such as MultiTEP aim to overcome these barriers by incorporating 12 universal non‐self T‐helper epitopes derived from common antigens, including tetanus toxoid, diphtheria toxin and influenza haemagglutinin. These epitopes provide enhanced co‐stimulatory signals, activating residual functional T cells in the elderly and improving immunogenicity even under conditions of immunosenescence [65, 106]. Whether this strategy fully compensates for the broader deficits of the aged immune system, including impaired germinal centre reactions, reduced somatic hypermutation and blunted innate sensing, remains to be established in adequately powered clinical trials.

### 5.2 Antigen Complexity, Neuropathological Heterogeneity and Trial Design

Both Aβ and Tau exhibit considerable conformational heterogeneity, with multiple isoforms and aggregation states, complicating the selection of epitopes that selectively target pathological species without interfering with their physiological counterparts [107]. A related, often underappreciated source of variability lies not in the vaccine construct itself but in the neuropathological heterogeneity of the enroled population. Most community‐dwelling older adults who meet clinical and biomarker criteria for AD also harbour at least one additional pathology, such as cerebrovascular lesions, limbic‐predominant age‐related TDP‐43 encephalopathy (LATE‐NC) or Lewy body disease, and these co‐pathologies independently drive cognitive decline [108]. In a community‐based autopsy cohort specifically selected to meet typical eligibility criteria for anti‐amyloid treatment, 94% carried a pathological diagnosis of AD, yet most also had at least one further pathology independently associated with a faster rate of decline [109].

This represents a structural limitation for trials of Aβ‐directed or Tau‐directed active immunisation that rely on cognitive endpoints: even a vaccine that fully achieves its intended biological effect on the targeted antigen may show attenuated or absent cognitive benefit if a substantial proportion of the enroled population's decline is in fact driven by untargeted coexisting pathology. This consideration argues for routine screening for major co‐pathologies in the design of future trials and for a broader shift towards biological rather than purely cognitive primary endpoints.

The timing of intervention is equally critical. Anti‐Tau approaches appear more effective when administered during prodromal or preclinical stages, whereas amyloid removal at symptomatic phases may be insufficient to modify disease progression. Conventional cognitive endpoints are often late and insensitive; the integration of fluid biomarkers, including p‐Tau, NfL and GFAP, alongside PET imaging allows for earlier assessment of biological efficacy, enabling shorter, more targeted trials in high‐risk populations [110]. Personalised interventions based on genetic and biomarker profiles, such as APOE ε4 carriership, amyloid‐positive MCI or autosomal dominant mutation status, represent a key direction for preventive strategies [111, 112].

### 5.3 Peripheral Immune Stimulation and the Herpes Zoster Precedent

Consistent with the herpesvirus‐related considerations discussed above, epidemiological evidence suggests that peripheral immune stimulation through unrelated vaccines may confer indirect neuroprotection. A natural experiment exploiting the age‐based eligibility cut‐off for herpes zoster vaccination in Wales found approximately 3.5 percentage points fewer new dementia diagnoses over seven years among those eligible for vaccination in a cohort of over 280,000 individuals [113].

This population‐level effect is fundamentally different from the patient‐level effects reported for active anti‐Aβ vaccines such as ABvac40, which demonstrated a 53% relative reduction in the hazard of clinically meaningful cognitive worsening in a Phase II trial of approximately 124 patients with amnestic MCI or very mild AD [48]. The two findings are not directly comparable in magnitude: one is a population‐wide estimate of dementia diagnosis over seven years in a largely cognitively normal cohort; the other is an HR for cognitive decline over a shorter follow‐up in an already symptomatic population. The contrast is nonetheless instructive. The herpes zoster vaccine carries no AD‐specific antigen and produces its effect through a single, well‐tolerated immunisation in the general elderly population, whereas ABvac40 requires repeated dosing of a purpose‐built peptide–carrier conjugate in patients already meeting criteria for cognitive impairment. If even part of the herpes zoster vaccine's apparent effect reflects reduced recurrent viral reactivation and its downstream innate‐immune and neuroinflammatory consequences, upstream pathogen‐directed immunisation in cognitively normal individuals may represent a more scalable preventive strategy than disease‐specific active immunotherapy initiated after measurable cognitive impairment has already occurred, without diminishing the rationale for AD‐specific vaccines in individuals with established pathology.

In parallel, data‐driven pharmacoepidemiological studies have begun to reveal unexpected associations between commonly prescribed medications and dementia risk, highlighting the potential of large‐scale, real‐world datasets to uncover modifiable factors and repurposing opportunities [114]. Such approaches may complement biomarker‐guided vaccine strategies by identifying pharmacological or immunological modifiers of neurodegeneration, ultimately supporting a more integrative and personalised framework for AD prevention.

### 5.4 Multi‐Target Vaccination: The Duvax Platform and Preclinical Proof of Concept

Within this framework, multi‐target vaccine platforms emerge as one of the most promising directions. The flexibility of the MultiTEP system enables the combination of Aβ and Tau epitopes on DNA or mRNA vectors, and preclinical studies in bigenic mouse models have demonstrated that combined immunisation with AV‐1959R and AV‐1980R simultaneously reduced both amyloid and Tau pathology, normalised microglial morphology and improved cognitive performance beyond that achieved by either single‐target vaccine alone [61].

Duvax is the most advanced bivalent vaccine currently in development, combining the recombinant constructs AV‐1959R and AV‐1980R, each built on the MultiTEP backbone, formulated with the AdvaxCpG adjuvant to enhance humoral immunity while maintaining low inflammatory potential [61, 85]. In 5xFAD/Tau22 bigenic mice, four intramuscular doses (20 µg each) induced high antibody titres against both Aβ and Tau (55–4785 µg/mL), reduced insoluble Aβ_42_ by 32% and hyperphosphorylated Tau by 25%, normalised microglial morphology and improved spatial and recognition memory compared with single‐target immunisation [61]. In PS19 Tau‐transgenic mice, the vaccine decreased insoluble and p‐Tau S396 by approximately 41%, reduced astrocytic GFAP expression, and improved motor performance, with antibody titres ranging from 276 to 5086 µg/mL [82, 83].

Toxicology studies revealed no signs of encephalitis, microhaemorrhage, necrosis or immune infiltration in brain tissue, and the DNA‐encoded variant AV‐1959D showed no genomic integration or systemic dissemination. Comparable safety and immunogenicity were observed in aged cynomolgus macaques, where anti‐Tau IgG levels averaged approximately 232 µg/mL without T‐cell autoreactivity [57]. These results establish preclinical proof of concept for Duvax as a synergistic, multi‐target vaccine capable of inducing parallel, non‐interfering antibody responses against both Aβ and Tau (NCT07142278).

Clinical translation is proceeding stepwise. Only the Aβ‐targeting DNA vaccine AV‐1959D has thus far progressed to human evaluation: the first‐in‐human Phase I study (NCT05642429) is currently enrolling 48 participants with early AD, testing intradermal doses of 500–2000 µg, with completion anticipated in late 2026 [115]. If safety and immunogenicity are confirmed, Duvax could serve as a prototype for preventive, durable and cost‐effective immunisation strategies acting on the dual pathogenic cascade of amyloid and Tau, directly addressing the multifactorial pathophysiology of the disease [116].

### 5.5 Novel Delivery Routes and Emerging Combinatorial Approaches

Alternative routes of administration may further broaden the immunological repertoire available to AD vaccine strategies. Intranasal delivery in particular may enhance immune responses by stimulating both systemic and mucosal immunity while minimising systemic inflammatory risk. The proteosomal adjuvant Protollin—derived from bacterial outer membrane proteins and lipopolysaccharides and originally developed to boost mucosal vaccine responses—activates innate immune cells and promotes microglial‐mediated clearance of Aβ plaques, inducing balanced IgG/IgA responses in preclinical models [117]. In a Phase I study (NCT07187141) involving 16 patients with early AD, intranasal Protollin proved safe and activated monocyte phagocytosis while reducing inflammation and CD8^+^ T‐cell cytotoxicity, supporting progression to Phase II evaluation of amyloid clearance [118]. This approach may represent a well‐tolerated alternative to systemic administration, particularly relevant for elderly populations in whom injection‐site reactogenicity and compliance with repeated parenteral dosing may be limiting.

Beyond combining Aβ and Tau targets, future vaccination strategies could integrate anti‐neuroinflammatory and antioxidant approaches, aiming to modulate the full spectrum of pathological processes underlying neurodegeneration [103]. Modulation of the regulatory T‐cell compartment represents a further, largely unexplored lever in this context. In murine AD models, regulatory T cells limit neuroinflammation and support microglial homeostasis, and their pharmacological expansion has been shown to reduce amyloid pathology and improve cognitive performance [119], suggesting that future active immunisation protocols could be deliberately designed to favour regulatory T‐cell responses alongside antibody induction, rather than treating the two as competing outcomes.

### 5.6 Conclusions

The past two decades have transformed active immunotherapy for AD from a proof‐of‐concept hypothesis into a scientifically grounded, clinically advancing field. The lessons of AN1792—on the dangers of autoreactive T‐cell responses, on the inadequacy of amyloid clearance alone as a surrogate for cognitive benefit and on the critical importance of therapeutic timing—have been systematically translated into more sophisticated vaccine designs, adjuvant choices and trial frameworks.

However, fundamental challenges remain. Immunosenescence constrains the magnitude and durability of antibody responses in the very population most in need of protection. The conformational and neuropathological heterogeneity of the disease means that even biologically successful vaccines may produce attenuated cognitive signals in trials that do not adequately account for coexisting pathologies and are not anchored in sensitive biological endpoints. The emerging evidence that upstream, pathogen‐directed immunisation may confer indirect neuroprotection at the population level raises the possibility that the most scalable preventive strategy may not be a disease‐specific vaccine at all, but rather the optimisation of existing immunisation programmes in older adults.

The path forward requires integrating conformationally selective immunogens, TLR‐engaging adjuvants, multi‐target platforms, alternative delivery routes and biomarker‐guided patient selection into cohesive clinical programmes. Critically, it also requires a shift in the intervention window: the evidence consistently points towards preclinical and prodromal stages as the moment when immunotherapy can still alter, rather than merely reflect, the disease trajectory. If bivalent Aβ/Tau vaccines such as Duvax fulfil their preclinical promise in human trials and if regulatory frameworks evolve to support earlier, biomarker‐stratified prevention, active immunisation could become the most scalable and cost‐effective tool available for modifying the natural history of AD, not merely managing its symptoms but interrupting the pathological cascade at a point when neuronal rescue remains within reach.

## Funding

Partially supported by grants to A.C. from Piano Nazionale di Ripresa e Resilienza (PNRR), Project “SIS‐NET” ‐ ID S4‐ 01.P0001 (grant number MUR PE00000007); by PRIN 2022, project “NI‐PACS”, code 020146_23, (grant number Prot. 2022NJYHMC); and by Project “Dipartimenti Eccellenti MIUR” (PDEM) 2022.

## Conflicts of Interest

The authors declare no conflicts of interest.

## Data Availability

The authors have nothing to report.
